# Long-term environmental exposure of darkness induces hyperandrogenism in PCOS *via* melatonin receptor 1A and aromatase reduction

**DOI:** 10.3389/fcell.2022.954186

**Published:** 2022-10-24

**Authors:** Weiwei Chu, Shang Li, Xueying Geng, Dongshuang Wang, Junyu Zhai, Gang Lu, Wai-Yee Chan, Zi-Jiang Chen, Yanzhi Du

**Affiliations:** ^1^ Center for Reproductive Medicine, Ren Ji Hospital, School of Medicine, Shanghai Jiao Tong University, Shanghai, China; ^2^ Shanghai Key Laboratory for Assisted Reproduction and Reproductive Genetics, Shanghai, China; ^3^ The Chinese University of Hong Kong-Shandong University Joint Laboratory on Reproductive Genetics, School of Biomedical Sciences, The Chinese University of Hong Kong, Hong Kong SAR, China; ^4^ National Research Center for Assisted Reproductive Technology and Reproductive Genetics, the Key Laboratory for Reproductive Endocrinology of Ministry of Education, Shandong Provincial Key Laboratory of Reproductive Medicine, Center for Reproductive Medicine, Shandong Provincial Hospital, Shandong University, Jinan, China

**Keywords:** constant darkness, polycystic ovary syndrome, hyperandrogenism, melatonin receptor 1A, androgen receptor

## Abstract

Polycystic ovary syndrome (PCOS) is a common and complex disorder impairing female fertility, yet its etiology remains elusive. It is reported that circadian rhythm disruption might play a crucial role in PCOS pathologic progression. Here, in this research, we investigated the effect of environmental long-term circadian rhythm dysfunction and clarified its pathogenic mechanism in the development of PCOS, which might provide the targeted clinical strategies to patients with PCOS. Female SD rats were used to construct a circadian rhythm misalignment model with constant darkness (12/12-h dark/dark cycle), and the control group was kept under normal circadian rhythm exposure (12/12-h light/dark cycle) for 8 weeks. We measured their reproductive, endocrinal, and metabolic profiles at different zeitgeber times (ZTs). Different rescue methods, including melatonin receptor agonist and normal circadian rhythm restoration, and *in vitro* experiments on the KGN cell line were performed. We found that long-term darkness caused PCOS-like reproductive abnormalities, including estrous cycle disorder, polycystic ovaries, LH elevation, hyperandrogenism, and glucose intolerance. In addition, the expression of melatonin receptor 1A (*Mtnr1a*) in ovarian granulosa cells significantly decreased in the darkness group. Normal light/dark cycle and melatonin receptor agonist application relieved hyperandrogenism of darkness-treated rats*. In vitro* experiments demonstrated that decreased *MTNR1A* inhibited androgen receptor (*AR*) and *CYP19A1* expression, and *AR* acted as an essential downstream factor of *MTNR1A* in modulating aromatase abundance. Overall, our finding demonstrates the significant influence of circadian rhythms on PCOS occurrence, suggests that MTNR1A and AR play vital roles in pathological progression of hyperandrogenism, and broadens current treatment strategies for PCOS in clinical practice.

## Introduction

Polycystic ovary syndrome (PCOS) is a complex endocrine disorder characterized by oligo/anovulation, high androgen levels, polycystic ovaries, and the exclusion of related disorders (Rotterdam ESHRE/ASRM-Sponsored PCOS Consensus Workshop Group, 2004). Most PCOS patients are in accordance with the criteria, yet about 20% of patients exhibit regular ovulatory menstrual cycles and 10% of them have normal androgen levels. The prevalence of PCOS is 10–15% in reproductive females, and it has become the most common syndrome affecting not only reproduction but also metabolism (Azziz et al., 2016). Women with PCOS are at higher risk for some leading causes of morbidity and early mortality, such as type 2 diabetes, hyperlipidemia, and cardiovascular diseases (Ali, 2015). In addition to genetic susceptibility, environmental factors, especially circadian rhythm disruption has drawn much attention in PCOS occurrence investigation recently (Mereness et al., 2015; Simon et al., 2019).

A diurnal rhythm is one crucial aspect in various species. The circadian rhythm aligns numerous cellular, biochemical, physiological, and behavioral processes (Zheng et al., 2021). It is regulated by the master circadian pacemaker located in the suprachiasmatic nuclei (SCN) of the hypothalamus (Levi and Schibler, 2007; Zisapel, 2018). The SCN synchronize the circadian physiological and behavioral rhythms, including sleep and wakefulness, temperature, feeding, neuroendocrine, and autonomic effects, with the 24-h periodicity to match the environmental light/dark cycle, to orchestrate an appropriate internal temporal order. As the fundamental stimulus for tuning, external light entrains the SCN rhythm period, synchronizes with the environment, and inhibits melatonin synthesis as well. Circadian rhythm misalignment may contribute to the development of a wide range of disorders, including fertility impairment (Willis et al., 2019; Gombert et al., 2021).

Until now, some studies have revealed an intimate relationship between diurnal rhythm and PCOS. Adolescent girls with PCOS suffered from circadian misalignment characterized by later melatonin offset relative to both clock time and sleep timing. Another integrative review suggested that PCOS patients seem to be at higher susceptibility to suffer from circadian rhythm disruption since the abnormal melatonin level may get involved in the neuroendocrine pathology of PCOS (Barron, 2007). The differences observed in melatonin receptors, such as genotype variations in patients and decreased expression levels in animal models, suggested a nonnegligible association between circadian rhythm disorder and PCOS (Song et al., 2015; Basheer et al., 2018). Our previous research also revealed that long-term environmental exposure to darkness induced the glycol-metabolic and reproductive hallmarks of PCOS for the first time (Li et al., 2020). Here, in this study, we aim to identify the efficiency of the circadian interference-induced PCOS model and to find out the specific underlying mechanism.

Hyperandrogenism is the most consistent biochemical change in PCOS, presenting in about 60–80% female patients according to Rotterdam criteria (Rotterdam ESHRE/ASRM-Sponsored PCOS Consensus Workshop Group, 2004). Excessive androgen production by the ovaries is considered one possible initial factor of PCOS (Azziz et al., 2016). It is associated with a worse prognosis and a higher risk of metabolic and cardiovascular diseases (Daan et al., 2015). Androgen excess may contribute to reproductive and metabolic dysfunction in PCOS, which was proved by dihydrotestosterone (DHT)- and dehydroepiandrosterone (DHEA)-treated animal models (Manneras et al., 2007; Zhang et al., 2016). Other studies supported this view by revealing androgen-induced autophagy activation and various gene set dysregulations, including cell cycle and glucose metabolism, all of which were related to pathophysiology changes in PCOS (Lee et al., 2019; Li et al., 2019). Excess androgen could produce circadian misalignment *via* tissue-dependent effects on phase distribution, while continuous light exposure led to hyperandrogenism (Kang et al., 2015; Mereness et al., 2015). These findings indicated the possible interplay between androgen and timing systems in PCOS morbidities.

Herein, this study was designed to assess the underexplored effect of circadian rhythm dysfunction in PCOS occurrence and to find out underlying molecular mechanisms. At the same time, we provided feasible therapeutic strategies to improve clinical treatment effects of PCOS females suffering from biorhythm misalignment.

## Materials and methods

### Animal model construction and treatments

**FIGURE 1:**
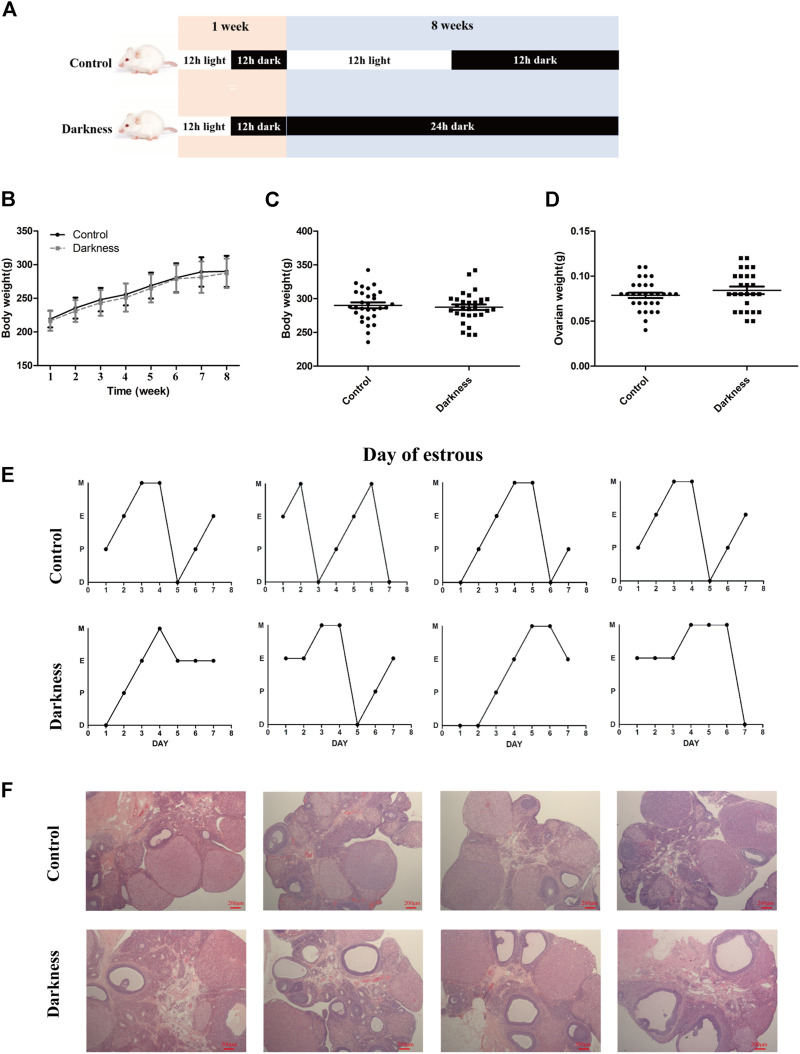
Long-term continuous darkness leads to estrous cycle disorder and polycystic ovary in rats. **(A)** Diagram of the experimental design. **(B,C)** Body weight of two groups of rats. **(D)** Ovarian weight of each group of rats. **(E)** Representative estrous cycles of two groups. The upper and lower panels represent the control and darkness groups, respectively. D, diestrus; P, proestrus; E, estrus; M, metestrus. **(F)** Representative examples of ovarian HE-stain histology of two groups. The upper and lower panels represent the control and darkness groups, respectively. *N* = 30/group. Values are expressed as means ± SEM. Scale bar: 200 μm.

Female SD rats aged 5 weeks were placed in constant temperature (21°C ± 2°C) with a humidity of 50% ± 5%, and food and water were provided *ad libitum*. All SD rats were acclimatized to a 12/12-h light/dark cycle for 1 week before subsequent experiments. A total of 60 SD rats were randomly divided into two groups: the control group and the darkness group (*N* = 30/group). The control group was kept under a 12/12-h light/dark cycle, and the darkness group was under a 12/12-h dark/dark cycle for eight consecutive weeks (Figure 1A). At the end of this experiment, five rats of each group were deeply anesthetized to sacrifice every 4 h from ZT0 (zeitgeber time) to ZT20. The ovaries and serum samples were collected and stored at −80°C.

As for the rescuing rat model construction, after 1-week acclimation, 30 female SD rats were randomly divided into six groups: control, darkness, ramelteon (RMT), rescue, control-10, and darkness-10 (*N* = 5/group). The control group and control-10 group were housed under a 12/12-h light/dark cycle, while the darkness group, darkness-10 group, and RMT group were housed under a 12/12-h dark/dark cycle for the indicated experimental duration. The RMT group was given RMT (8 mg/kg B.W./d, MedChemExpress, New Jersey, United States) by gavage at ZT12. The rescue group was kept under a 12/12-h dark/dark cycle for 8 weeks, followed by a normal 12/12-h light/dark cycle for 2 weeks (Figure 5A). At the end of the experiment, rats of each group were anesthetized to sacrifice at ZT0.

Body weight was measured once a week throughout the experiment. Estrus cycles were monitored during the last eight consecutive days and identified using a microscope (Zeiss, Oberkochen, Germany) as previously described (Marcondes FK et al., 2002).

All of the experimental protocols were carried out in accordance with institutional guidelines and approved by the Ethics Committee of Shanghai Jiao Tong University School of Medicine.

### Glucose tolerance test

Following an overnight fasting for 16 h, 50% d-glucose solution (2 g/kg B.W.) was injected intraperitoneally. Blood samples of rats were obtained from the tail vein, and the glucose levels were measured at indicated times using an Accu-Chek glucose monitor (Roche, Basel, Switzerland).

### Reproduction profile and biochemical indexes

Trunk circulation blood samples were collected and placed still at room temperature for 2 h. Then, serum samples were obtained after centrifuging at 2500 rpm for 15 min. Luteinizing hormone (LH), follicle-stimulating hormone (FSH), and sex hormone-binding globulin (SHBG) levels were measured using enzyme-linked immunosorbent assay (ELISA) kits (MyBioSource, San Diego, United States) using a microplate reader (Tecan, Swiss). Total testosterone, estradiol, and melatonin were indicated with ELISA kits purchased from Cayman Chemical (Michigan, United States), R&D Systems (Minneapolis, United States), and Cusabio Technology LLC (Wuhan, China), respectively. All the procedures were carried out according to standard manufacturers' protocols.

### Histology

For hematoxylin and eosin (HE) staining, rat ovaries were fixed in 4% paraformaldehyde and embedded in paraffin, which were cut through the maximal axis at 5 μm thickness. The sections were deparaffinized and rehydrated through a graded ethanol series, stained in hematoxylin and differentiated by hydrochloric acid, and finally incubated in eosin before covering the slides. Image visualization was proceeded using a microscope (Zeiss, Oberkochen, Germany).

For immunohistochemistry (IHC), ovary sections were blocked with BSA, incubated with MT1 (protein of *MTNR1A*) antibody (Abcam, Cambridge, United Kingdom) at 1:200 dilution overnight at 4°C in a dark room and then incubated with the secondary antibody. Diaminobenzidine (DAB) was applied for the color reaction. All slides were imaged for 10 random fields at ×20 magnification using a microscope (Zeiss, Oberkochen, Germany) and then analyzed using Image-Pro Plus 6.0.

### Cell culture and treatments

The KGN human granular carcinoma cell line was cultured in DMEM/F12 (Gibco, NY, United States) containing 10% charcoal-stripped fetal bovine serum (Thermo Fisher Scientific, MA, United States) and 1% antibiotics (mixture of penicillin, streptomycin, and neomycin) (Gibco) in an incubator at 37°C and 5% CO_2_ (Thermo Fisher Scientific). The cells were passaged every 2–3 days.

For FSH stimulation, the cell culture medium was replaced with a fresh medium containing 10 ng/ml recombinant FSH (rFSH, MyBioSource Inc.) as previously described (Foldesi et al., 1998). To investigate the activity of the androgen receptor (AR) and melatonin receptor 1A (MTNR1A), 10 nmol/L testosterone (R&D Systems) or 10 nmol/L melatonin (Sigma-Aldrich, MO, United States) was supplemented in a fresh medium as the substrates. In addition, cAMP (Sigma-Aldrich) of different concentrations was added to the fresh medium to promote aromatase function.

### Cell transfection

KGN cells were seeded into six-well plates and cultured for 24 h. After culture medium refreshing, the cells were transfected with siRNAs (GenePharma, Shanghai, China) or pCMV3 plasmid with the *AR* clone (NM_000044.6) (Transheep, Shanghai, China) with Lipofectamine 3000 (Invitrogen, CA, United States) according to the manufacturer’s instructions. Negative control siRNA and blank vectors were provided by GeneChem (Shanghai, China). Afterward, KGN cells were incubated for 48 or 72 h, followed by further treatments. The specific siRNA sequences of the target genes were as follows:

*MTNR1A* siRNA, 5′-GGG​UGA​AAC​CUG​ACC​GCA​ATT-3’; AR siRNA, 5′- CUG​CUA​CUC​UUC​AGC​AUU​ATT-3’.

### RNA extraction and qRT-PCR

Total RNA was extracted using an RNA kit (FOREGENE, Chengdu, China) according to the manufacturer’s instructions. The RNA concentration and quality were measured using NanoDrop ND-2000 (Thermo Scientific). Then, isolated RNA was reverse-transcribed into cDNA (PrimeScript RT Master Mix, Takara, Dalian, China). Target gene expression was measured through qRT-PCR with SYBR Premix Ex Taq (Takara). Subsequently, the 2^-△△Ct^ method was used to analyze relative mRNA levels normalized to β-actin. The primer sequences of target genes are listed in Supplementary Table S1.

### Protein extraction and Western blotting

Total proteins were extracted using ice-cold radioimmunoprecipitation assay lysis buffer (Shenggong, Shanghai, China), supplemented with protease and phosphatase inhibitors (Roche, Basel, Switzerland). Equal amounts of protein were denatured at 100°C for 10 min and then electrophoresed in 10% SDS-polyacrylamide gels. Afterward, blots were wet-transferred to nitrocellulose membranes (Millipore, Billerica, MA). After blocking, blots were incubated with primary antibodies against MT1 (Abcam, Cambridge, United Kingdom), AR, and aromatase (both from Santa Cruz Biotechnology, United States) at 1:600–1:1000 dilutions and then incubated against secondary antibodies. An enhanced chemiluminescent detection system (Millipore) was used to detect bands with peroxidase activity. The same blot was probed with GAPDH (Proteintech, Chicago, United States) as the internal loading control. The bands were visualized using a G-Box iChemi Chemiluminescence Image Capture System (Syngene, Frederick, MD, United States).

### Human subject recruitment and granulosa cell collection

Women participated in this research were recruited from patients undergoing *in vitro* fertilization–embryo transfer (IVF-ET) in the Center for Reproductive Medicine, Ren Ji Hospital, Shanghai Jiao Tong University School of Medicine. Appropriate informed consent was obtained from all patients enrolled in this study. A total of 28 patients who fulfilled all three of the Rotterdam criteria were recruited into the PCOS group (Rotterdam ESHRE/ASRM-Sponsored PCOS Consensus Workshop Group, 2004), and 42 women with tubal factor and/or male factor-related infertility were enrolled into the non-PCOS group. All subjects were of Han ethnicity in reproductive age and had not received hormonal therapy for 3 months until recruitment. The clinical information of participants is presented in Supplementary Table S2.

The same GnRH antagonist protocol with hCG trigger was applied for ovarian stimulation and oocyte retrieval in both non-PCOS and PCOS groups. On the retrieval day, follicular fluid was collected from the size-matched dominant follicles with a diameter of approximately 18–20 mm, and human granulosa cells (hGCs) in follicular fluid were isolated as previously described using Ficoll-Paque™ PLUS (GE Healthcare Bio-Science, Uppsala, Sweden) and hyaluronidase (Sigma-Aldrich) (Iwase et al., 2009). Finally, hGCs from a single subject were isolated for RNA extraction and further qRT-PCR. All the experiment procedures were reviewed and approved by the Institutional Review Board of Ren Ji Hospital, School of Medicine, Shanghai Jiao Tong University.

### Statistical analysis

The results are presented as mean ± SEM. Each experiment was repeated 3 to 5 times. The data were initially subjected to Kolmogorov–Smirnov tests to assess deviation from the Gaussian distribution. For normally distributed data, paired Student’s t-test or one-way analysis of variance (ANOVA) followed by the Newman–Keuls multiple comparison test was performed. For data not normally distributed, we applied the Kruskal–Wallis test followed by Dunn’s multiple comparison test. For all tests, a two-tailed *p*-value < 0.05 was considered statistically significant. Statistical significance is shown as *p < 0.05, **p < 0.01, and ***p < 0 0.001.

## Results

### Long-term darkness caused reproductive hallmarks of PCOS in SD rats

To validate the contribution of circadian rhythm disorder to PCOS occurrence, we built up the rat model with constant darkness treatment for eight consecutive weeks (Figure 1A). We examined the relative mRNA expression of the circadian gene in the ovaries of each group of rats. As shown in Supplementary Figure S1, the mRNA expression profile of circadian genes, such as *Bmal1* and *Per1*, was different between the two groups. Constant darkness disrupted the normal estrous cycles of control rats (Figure 1E), while there were no differences in body weight or ovarian weight between the two groups **(** Figures 1B–D). In addition, there were more follicles with the presence of corpus luteum in rat ovaries of the control group, while the darkness group of rats harbored more abnormal enlarged follicular cysts (Figure 1F). These results suggested that constant darkness had negative effects on ovarian reproductive function in rats.

### Long-term darkness led to endocrinal and glucose metabolic abnormality in SD rats

**FIGURE 2:**
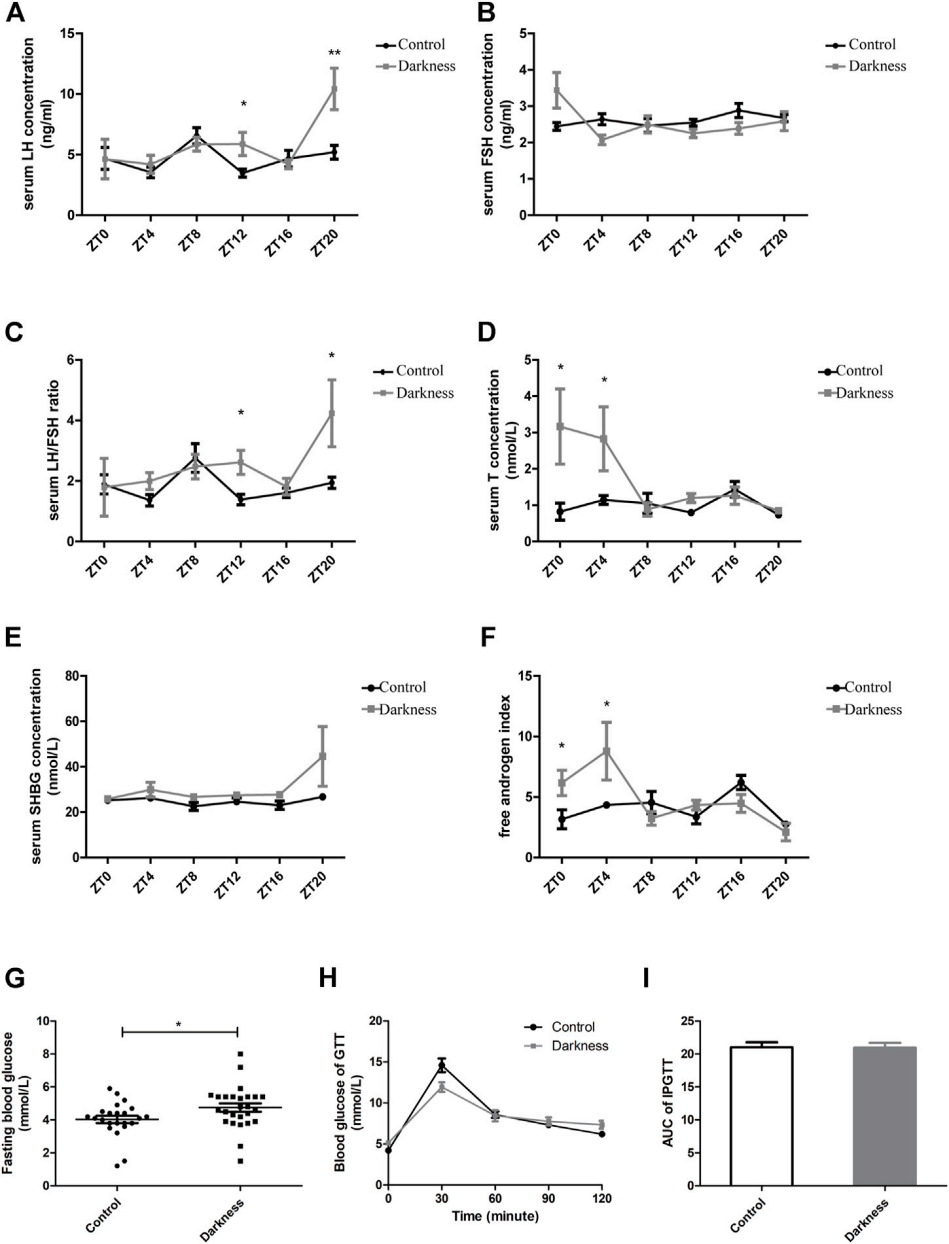
Serum hormone profiles and glucose metabolism of rats. **(A)** Serum concentration of LH. **(B)** Serum concentration of FSH. **(C)** LH/FSH ratio. **(D)** Serum concentration of testosterone. **(E)** Serum concentration of SHBG. **(F)** Free androgen index calculated by total testosterone plus 100 and divided by SHBG. **(G)** Fasting blood glucose concentration. **(H)** Glucose tolerance test. **(I)** Area under the curve of the GTT test. *N* = 5/group. Values are expressed as means ± SEM. Significant differences between the two groups are indicated by asterisks (*p < 0.05 and **p < 0.01).

Significant variations in serum hormone and fasting blood glucose were observed between rats of control and darkness groups. After treatment for 8 weeks, the serum LH level and LH/FSH ratio were much higher in the darkness group of rats at ZT12 and ZT20 (Figures 2A,C), but serum FSH remained unchanged (Figure 2B). Also, we found that the total testosterone (T) level increased at ZT0 and ZT4 (Figure 2D), and sex hormone-binding globulin (SHBG) was identical (Figure 2E). Thus, the free androgen index was elevated at ZT0 and ZT4 (Figure 2F) in the darkness group of rats. In addition, continuous darkness caused an increased level of fasting blood glucose (Figure 2G). The area under the curve (AUC) of the glucose tolerance test (GTT) was not variant between the two groups (Figures 2H,I **)**. Based on these findings, we concluded that constant darkness would induce PCOS-like reproductive and metabolic changes in rats.

### Constant darkness reduced the expression of *Cyp19a1* and *Ar* in rat ovarian GCs

**FIGURE 3:**
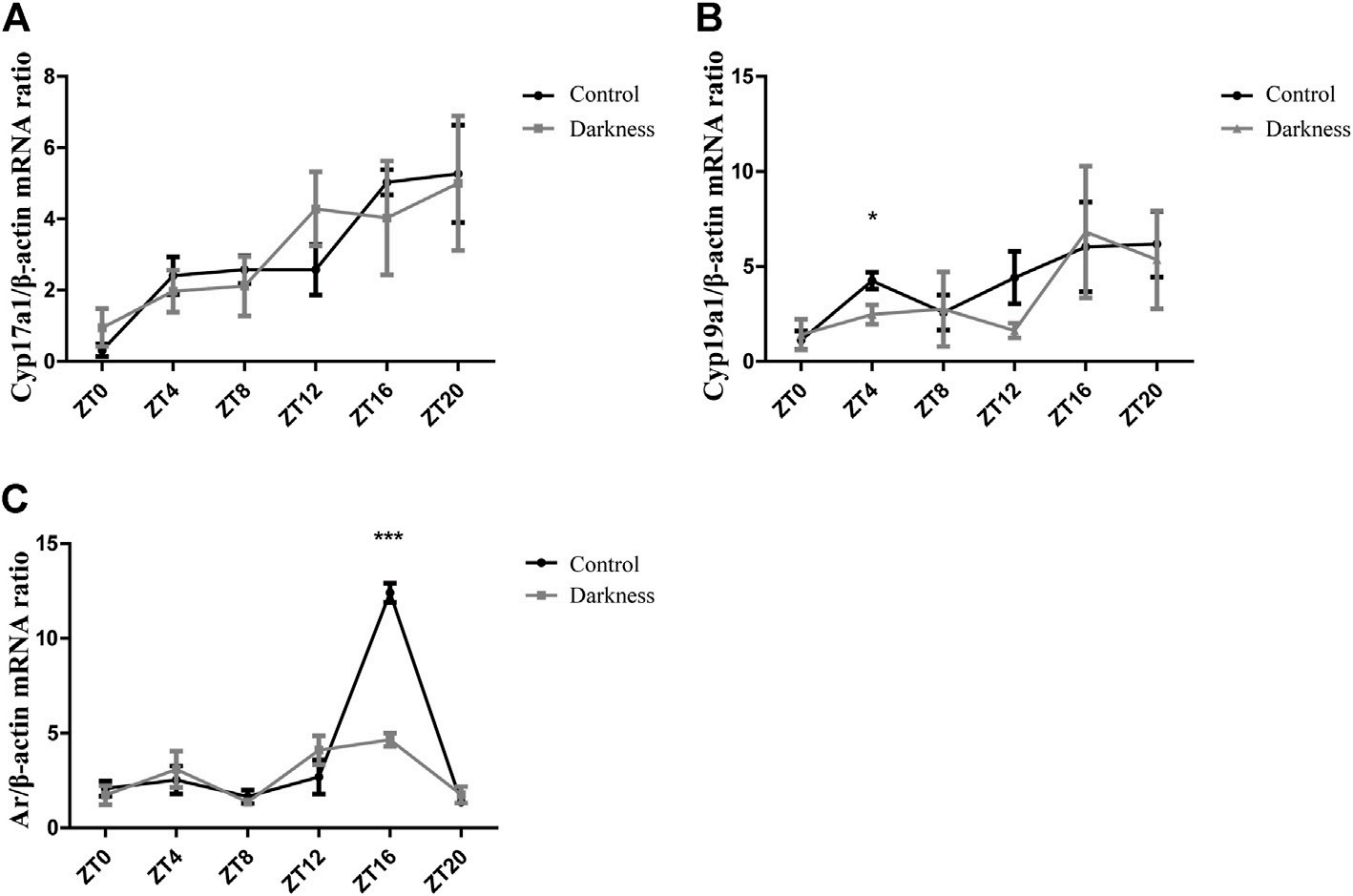
Gene expression patterns of *Cyp17a1*, *Cyp19a1*, and *Ar* in rat ovaries. **(A)** Relative mRNA abundance of *Cyp17a1* in rat ovaries. **(B)** Relative mRNA abundance of *Cyp19a1* in rat ovaries. **(C)** Relative mRNA abundance of *Ar* in rat ovaries. *N* = 5/group. Values are expressed as means ± SEM. Significant differences between the two groups are indicated by asterisks (*p < 0.05, **p < 0.01, and ***p < 0.001).

To investigate the possible mechanism of hyperandrogenism in the darkness group, we examined the mRNA abundance of steroid hormone biosynthesis enzymes in rat ovaries.According to the two-cell, two-gonadotrophin hypothesis of steroidogenesis in the human ovary, *CYP17* gene encoding 17-hydroxylase/C17-20-lyase activity, which is crucial to androgen synthesis, is expressed exclusively in thecal cells. GCs undertake LH-responsive aromatization and convert androgen to estrogen (Hillier SG et al., 1994). In this study, we found that the mRNA expression of *Cyp17a1* was identical between the two groups of rats (Figure 3A), while the relative abundance of *Cyp19a1*, which encodes aromatase, was significantly lower at ZT4 (Figure 3B) in the darkness group of rats than control ones.

On the other hand, granulosa cells also express AR, and theca-derived androgen has the potential to modulate locally differentiative responses to FSH (Hillier SG et al., 1994). In this study, we measured the mRNA level of *Ar* in ovaries and found it to be much lower at ZT16 in the darkness group (Figure 3C). Based on the aforementioned results, androgen excess in the darkness group possibly resulted from the low expression of aromatase in the ovaries, and *Ar* might get involved in this pathophysiologic process.

### Circadian rhythm misalignment caused downregulation of MT1 in rat ovarian GCs

**FIGURE 4:**
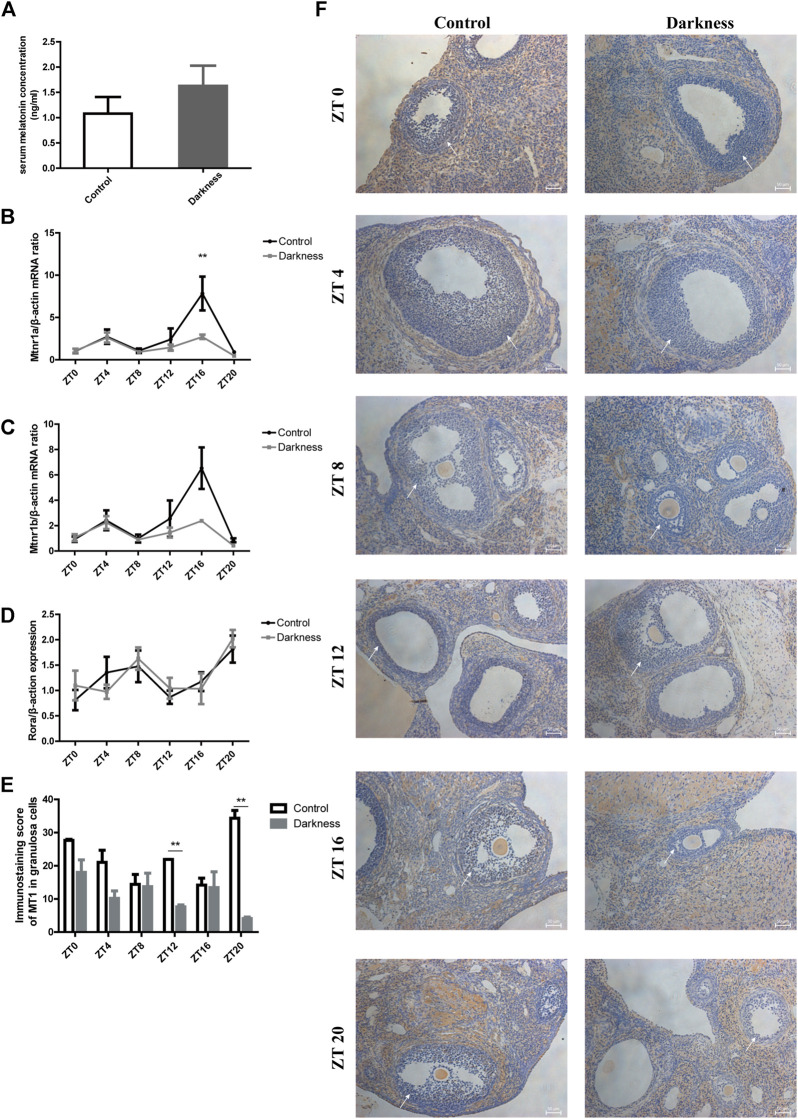
Melatonin level in serum and its receptor expression in rat ovaries. **(A)** Serum level of melatonin at ZT16. **(B)** Relative mRNA abundance of *Mtnr1a* in rat ovaries. **(C)** Relative mRNA abundance of *Mtnr1b* in rat ovaries. **(D)** Relative mRNA abundance of *Rora* in rat ovaries. **(E)** Immunostaining score of MT1 in ovarian GCs of rats. **(F)** Representative photomicrographs of MT1 in follicular GCs. Scale bar: 50 μm. *N* = 5/group. Values are expressed as means ± SEM. Significant differences between the two groups are indicated by asterisks (*p < 0.05 and **p < 0.01).

To study the effect of circadian rhythm disorder on melatonin and its receptors, we detected their expression in ovaries. The serum melatonin concentration was not varied between the two groups (Figure 4A). Receptors of melatonin in rat ovaries include membrane receptors such as *Mtnr1a*, melatonin receptor 1 b (*Mtnr1b*), and nuclear receptor *Rora*. In this study, we found decreased mRNA abundance of *Mtnr1a* at ZT16 in the darkness group of rats (Figure 4B). There were no differences in *Mtnr1b* or *Rora* expression between the two groups (Figures 4C,D).

To further validate the role of *Mtnr1a* in circadian rhythm disorder, we detected the location and abundance of MT1 through immunohistochemical analysis. As shown in Figure 4F, MT1 was expressed in GCs, theca cells, and stroma in rat ovaries. In accordance with the previous finding in Figure 4B, we found that the immunostaining score of MT1 was significantly lower at ZT12 and ZT20 in the darkness group (Figure 4E). These results indicated that constant darkness caused downregulation of MT1 in ovarian GCs of rats.

### Rescue treatment alleviated reproductive abnormalities in long-term darkness-treated rats

**FIGURE 5:**
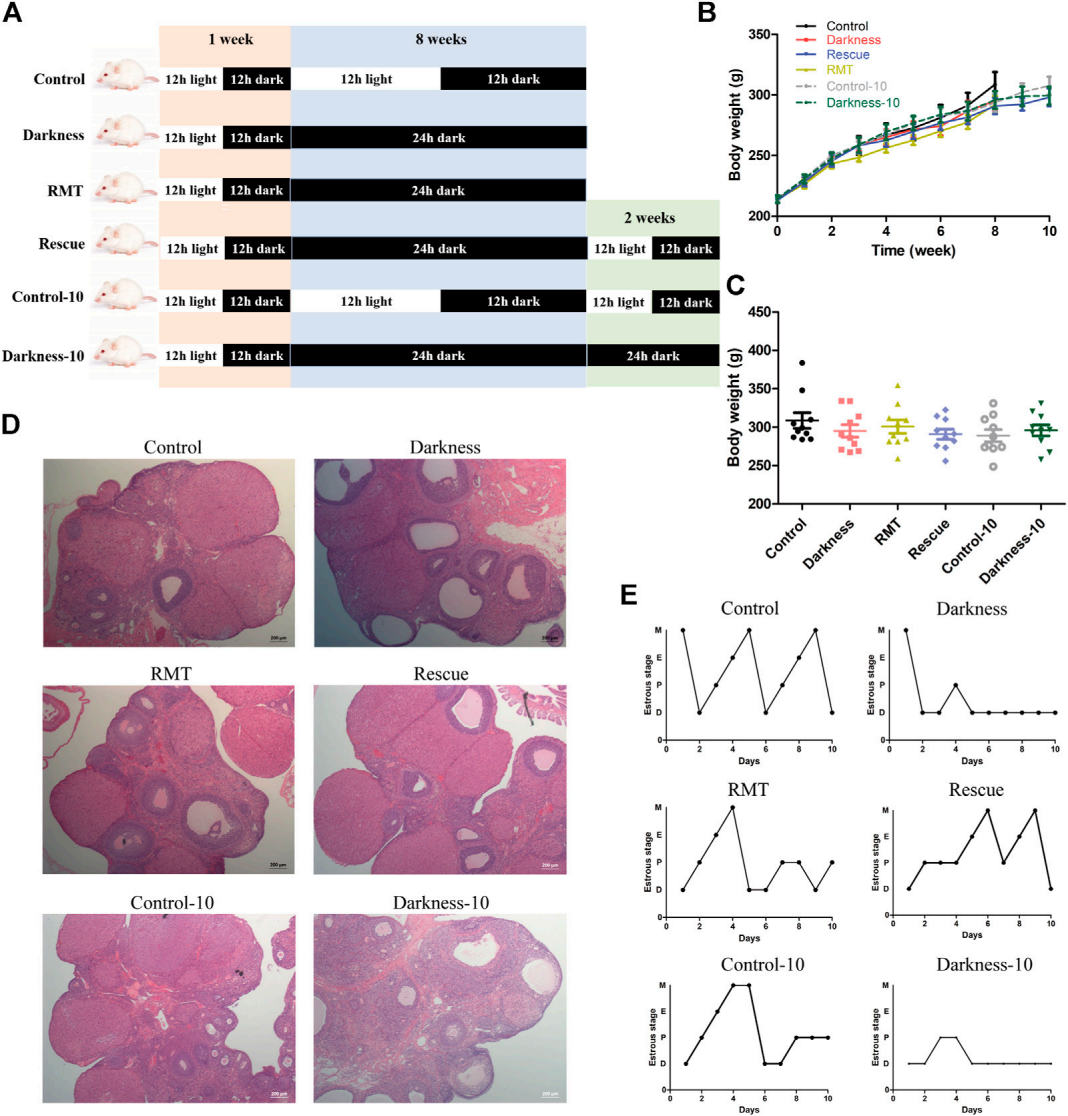
Effects of RMT and circadian rhythm restoration in constant darkness induced-PCOS rats. **(A)** Diagram of the experimental design. **(B,C)** Body weight of rats during the experimental progression. **(D)** Representative examples of ovarian HE-stain histology of each group of rats. **(E)** Representative estrous cycles of each group of rats. *N* = 10/group. Values are expressed as means ± SEM. Scale bar: 200 μm. RMT, ramelteon.

To investigate practical therapeutic methods to improve PCOS-like alterations and validate variation of *Mtnr1a* in the progress, we applied melatonin receptor agonist RMT and restored the normal light/dark circadian rhythm. To exclude that the constant darkness-induced manifestation might be relieved spontaneously as time went by, prolonged experimental duration of 10-week treatment served as another control (Figure 5A). Similarly, the body weights among different groups of rats were identical (Figures 5B,C). Both the darkness group and darkness-10 group of rats harbored abnormally enlarged follicles, less corpora luteum (Figure 5D), and less estrus stage (Figure 5E) compared to the control group and control-10 group. The polycystic ovary and estrous disruption were partially alleviated with RMT application or circadian rhythm restoration.

### Rescue treatment improved endocrinal hormone imbalance in the darkness group of rats

**FIGURE 6:**
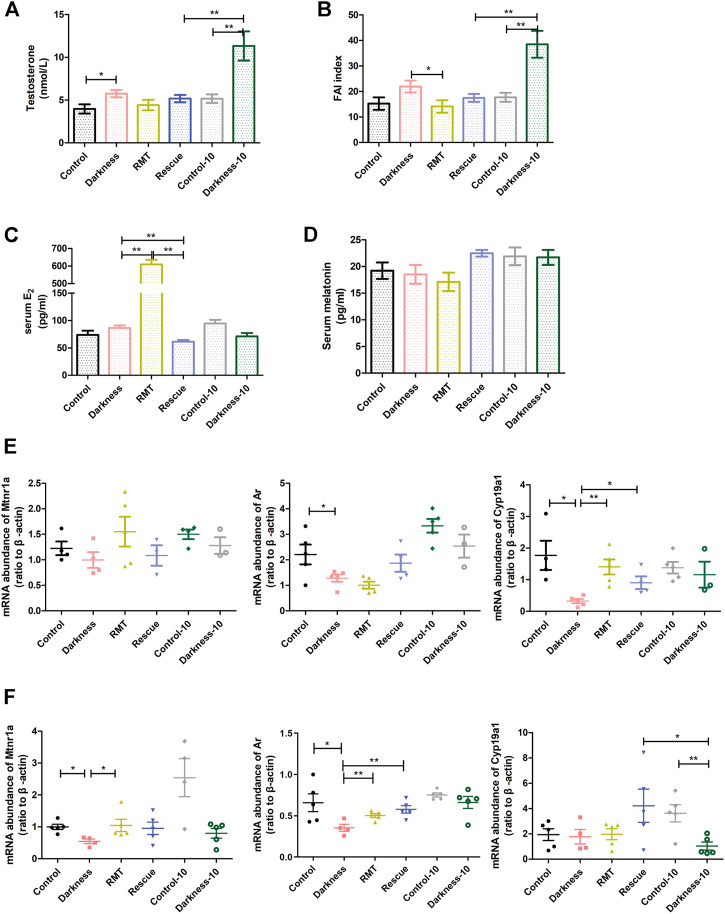
Influences of RMT and circadian rhythm restoration on the reproductive endocrine system and gene expression abundance. **(A)** Serum concentration of testosterone. **(B)** Free androgen index. **(C)** Serum estradiol content. **(D)** Serum concentration of melatonin. **(E)** *Mtnr1a*, *Ar*, and *Cyp19a1* mRNA levels in ovarian GCs of each group of rats. **(F)** *Mtnr1a*, *Ar*, and *Cyp19a1* mRNA levels in whole ovaries of each group of rats. *N* = 5/group. Values are expressed as means ± SEM. Significant differences between the two groups are indicated by asterisks (*p < 0.05 and **p < 0.01).

Rats enduring light deprivation for 8 and 10 weeks displayed hyperandrogenism, whereas restoring light/dark exposure decreased the T level compared with the darkness-10 group of rats (Figure 6A). Although there were no differences in SHBG among groups of rats (Supplementary Figure S2A), melatonin receptor agonist application reduced the free androgen index (FAI) apparently (Figure 6B). Restoring the light/dark cyclic rhythm also alleviated high FAI compared with the darkness-10 group (Figure 6B). In addition, the RMT group of rats showed a remarkably higher level of estradiol (Figure 6C), suggesting more androgen conversion activity after melatonin receptor agonist treatment.

To decipher the mechanism of rescue treatment on circadian rhythm disorder, we measured the serum level of melatonin and detected mRNA expression of melatonin receptors, *Ar*, and *Cyp19a1* in rat ovaries. Neither constant darkness nor two rescue treatment methods caused alterations in serum melatonin levels (Figure 6D). To better understand the influence of the rescue treatment, we extracted the GCs of rat ovaries and detected the gene expression levels. Constant darkness did not affect the expression level of *Mtnr1a* (Figure 6E), *Mtnr1b*, or *Rora* (Supplementary Figure S2B). But light deprivation for 8 weeks caused a decreased abundance of *Ar* and *Cyp19a1* in ovarian GCs (Figure 6E). Both RMT application and rhythm restoration alleviated *Cyp19a1* reduction in GCs (Figure 6E). Owing to various kinds of cell existence in the ovary, the expression of the molecules was distinguished to some extent. The mRNA abundance of *Mtnr1a* was significantly lower in the rat ovaries of the darkness group than the control group (Figure 6F). The expression of *Mtnr1b* and *Rora* in ovaries was identical after the treatment of constant darkness (Supplementary Figure S2C). RMT treatment improved the reduction of *Mtnr1a* and *Ar* in rat ovaries resulting from constant darkness, while rhythm restoration alleviated *Cyp19a1* reduction compared with the darkness-10 group (Figure 6F).

### Knockdown of *MTNR1A* and *AR* decreased aromatase expression *in vitro*

**FIGURE 7:**
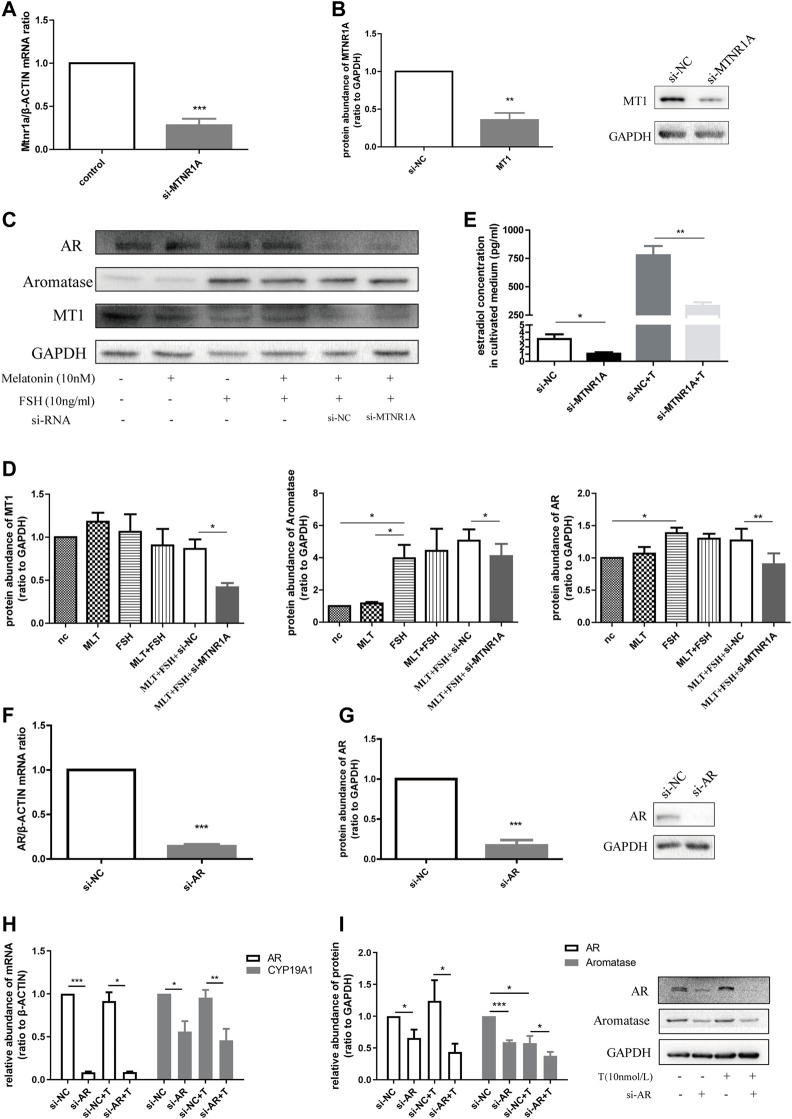
Downregulated *MTNR1A* and *AR* reduced aromatase expression in the KGN cell line and decreased estradiol concentration in the culture medium. **(A,B)** Efficiency of *MTNR1A-*knockdown in mRNA and protein levels, respectively. **(C,D)** Western blot analysis **(C)** and quantification **(D)** of AR, aromatase, and MT1 after *MTNR1A*-knockdown and incubation with or without 10 nmol/L melatonin or 10 ng/ml FSH. **(E)** Estradiol level after *MTNR1A*-knockdown and incubation with or without 10 nmol/L testosterone. **(F,G)** Efficiency of *AR*-knockdown in mRNA and protein levels, respectively. **(H,I)** mRNA and protein abundance of *AR* and *CYP19A1* after *AR*-knockdown and incubation with or without 10 nmol/L testosterone, respectively. Values are expressed as means ± SEM. These results are representative of at least three independent experiments. Significant differences between the two groups are indicated by asterisks (*p < 0.05, **p < 0.01, and ***p < 0.001).

To decipher the mechanism by which *MTNR1A* contributed to androgen excess, we made use of human granulosa-like KGN cells in this experiment. Figures 7A,B demonstrate the knockdown efficiency of *MTNR1A* in mRNA and protein levels. To evaluate the function of *MTNR1A*, KGN cells were exposed to *MTNR1A*-siRNA with rFSH and melatonin in the culture medium (Figure 7C). It showed that the knockdown of *MTNR1A* led to lower protein expression of MT1, aromatase, and AR (Figure 7D). Then, 10 nmol/L testosterone was added to the cell culture medium to examine the effect of *MTNR1A* in the androgen conversion process. Through culture medium examination, we found that the knockdown of *MTNR1A* caused decreased androgen conversion with or without testosterone in the culture medium (Figure 7E).

Next, to know about the effect of *AR* on aromatase expression, we treated the KGN cell line with *AR*-siRNA and examined the knockdown efficiency in mRNA and protein levels (Figures 7F,G). Similarly, treatment with *AR*-siRNA caused decreased mRNA abundance of *CYP19A1*, and *AR*-knockdown with testosterone addition brought about a lower mRNA level (Figure 7H). The protein expression of aromatase decreased after *AR*-knockdown with testosterone in the medium (Figure 7I). Taken together, we summarized that *MTNR1A* regulated *AR* and aromatase expression, namely, androgen conversion in KGN cells. Decreased *MTNR1A* could reduce *AR* expression, resulting in less aromatase abundance and androgen conversion. *AR* could be a downstream factor of *MTNR1A* in the abnormal aromatase synthesis process due to circadian rhythm misalignment.

#### *MTNR1A* regulated *AR* and *CYP19A1* expression through the cAMP/PKA signaling pathway

**FIGURE 8:**
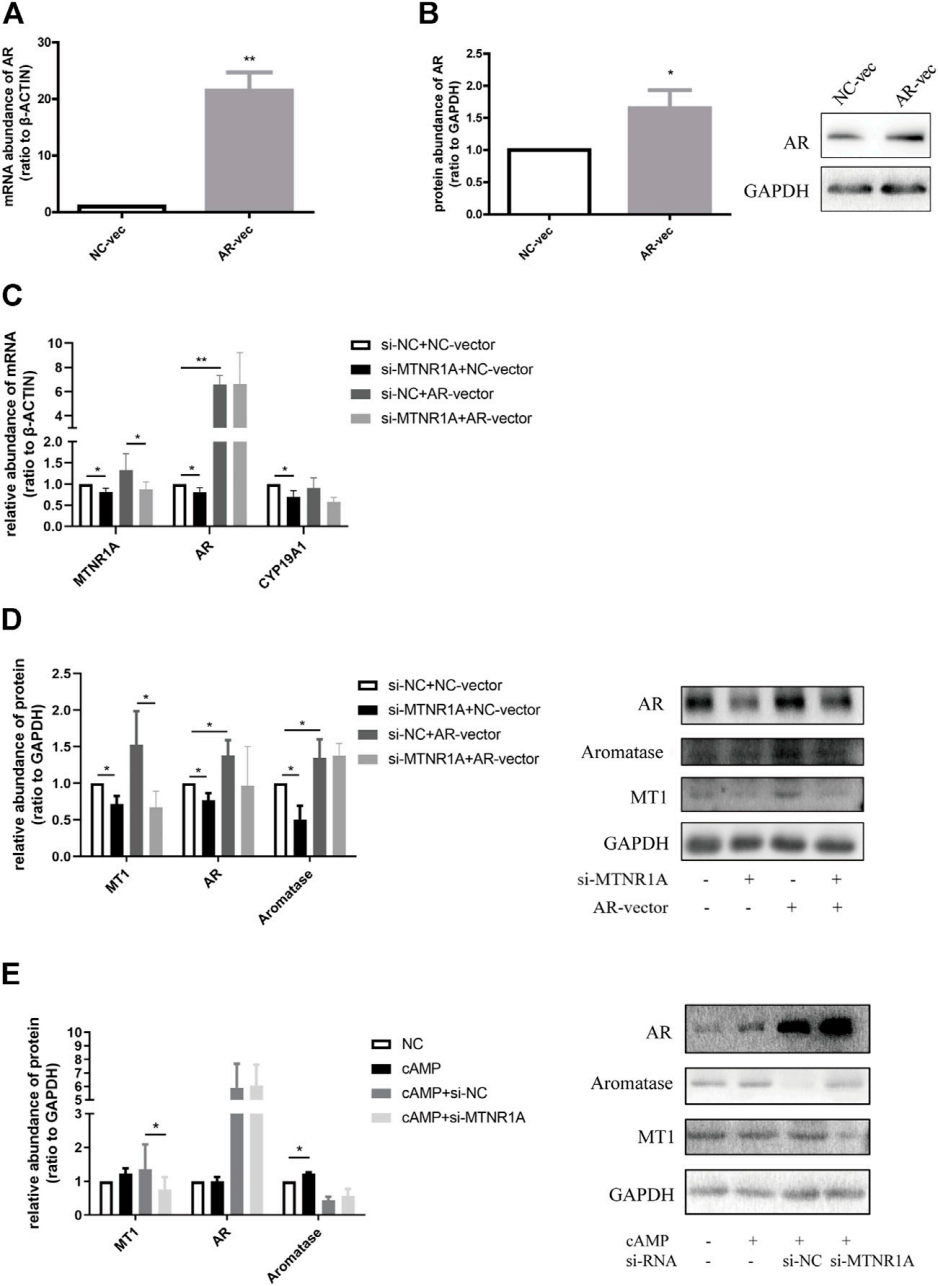
*MTNR1A* regulated aromatase expression through *AR* and PKA/cAMP pathway. **(A,B)** Efficiency of *AR*-overexpression in mRNA and protein levels, respectively. **(C,D)** mRNA and protein abundance of *MTNR1A*, *AR*, and *CYP19A1* after *MTNR1A*-knockdown and *AR*-overexpression, respectively. **(E)** Protein abundance of MT1, AR, and aromatase after *MTNR1A-*knockdown and incubation with 100 μmol/L cAMP for 24 h. Values are expressed as means ± SEM. These results are representative of at least three independent experiments. Significant differences between the two groups are indicated by asterisks (*p < 0.05 and **p < 0.01).

To know about the effect of *AR* in *CYP19A1* regulation, we overexpressed *AR* through vector transfection and examined the efficiency in mRNA and protein levels (Figures 8A,B). Similar to previous findings, mRNA and protein abundance of *CYP19A1* decreased after treatment with *MTNR1A*-siRNA, and *AR*-overexpression caused higher protein abundance of aromatase (Figures 8C,D). Of note, the expression of aromatase in the si-*MTNR1A* + *AR*-vector group was not decreased compared with that of the si-NC + *AR*-vector group (Figures 8C,D), demonstrating that *AR* acts as a vital downstream factor of *MTNR1A* in the *CYP19A1* regulation process.

Previously, numerous evidence showed that melatonin could bind to its membrane receptor MT1 and function *via* the cyclic adenosine monophosphate/protein kinase A (cAMP/PKA) signaling pathway (Chen et al., 2014; Tao and Zhu, 2018). After examining the appropriate cAMP concentration to activate and promote aromatase production (Supplementary Figure 3), we examined the effect of *MTNR1A*-siRNA treatment with 100 μmol/L cAMP. As shown in Figure 8E, cAMP treatment increased the protein abundance of aromatase compared with the NC group. However, with cAMP incubation, the knockdown of *MTNR1A* did not affect the expression of AR or aromatase compared with the si-NC group (Figure 8E). Based on the existing results, we concluded that *MTNR1A* regulated aromatase expression through the cAMP/PKA signaling pathway, and *AR* is a downstream of *MTNR1A* in this pathological process.

### Decreased expression of *MTNR1A* and *CYP19A1* in PCOS patients

To confirm this variation in women with PCOS, we recruited subjects who attended the Reproductive Clinical Center and collected the hGCs during the oocyte retrieval process. Compared with the non-PCOS group, the PCOS group was characterized by higher body mass index and AMH, as well as increased basal LH, testosterone, and fasting insulin levels (Supplementary Table S2). In addition, mRNA levels of *MTNR1A* and *CYP19A1* in hGCs were significantly lower in the PCOS group (Supplementary Figure S4). This finding was in accordance with changes in the PCOS-like rat model, thus validating the important role of melatonin receptors in the occurrence of hyperandrogenism in PCOS patients.

## Discussion

PCOS is the most common disorder interfering female reproduction and metabolism. Figuring out the cause of PCOS would help to resolve much difficulty in clinical practice. The circadian rhythm aligns numerous cellular, biochemical, physiological, and behavioral processes, including cell division, hormone secretion, sleep–wake cycle, and reproduction (Zheng et al., 2021). These oscillations are driven by abiotic environmental changes such as light and temperature. It is known to all that dysregulation of circadian rhythms can interfere human health and fitness, but its consequences in female reproduction, such as PCOS, could still be underestimated. In our previous research, we demonstrated that arrhythmic expression of circadian clock genes caused glycol-metabolic and reproductive hallmarks of PCOS first (Li et al., 2020). Here, in the present study, to find out the relationship between circadian rhythm misalignment and hyperandrogenism in PCOS, we constructed the animal model and explored its consequences following this environmental aspect.

In this study, we deprived light exposure for consecutive 8 weeks to remove the effect of external 24-h periodic rhythm. We observed estrous cycle disorder and polycystic ovarian malformation in the darkness group of rats. In addition, the endocrinal hormonal profiles were obviously different between the darkness and control groups of rats. Under specific zeitgeber time, serum levels of LH, total testosterone, LH/FSH ratio, and FAI were significantly higher in the darkness group. The fasting blood glucose concentration increased after continuous light deprivation, too. These results demonstrated constant darkness, an abnormal treatment of circadian rhythm misalignment, resulted in PCOS-like morbidity changes. The abnormal expression of circadian genes in rat ovaries also indicated an important role of circadian rhythm in the occurrence of PCOS.

Melatonin is a hormone synthesized by the pineal gland. Its circadian rhythm is regulated by the SCN; thus, melatonin always acts as a marker of SCN timing. Due to bright light acutely inhibiting melatonin production, the content of melatonin is low during the daytime, elevates in the evening, and returns to lower daytime levels during habitual waketime (Zeitzer et al., 2000). In this study, we measured the melatonin level in serum but found no difference between the two groups of rats. Contradictory to our finding, Farhadi et al. found that serum melatonin significantly increased after constant darkness for 10 days (Farhadi et al., 2016). We suppose that the inconsistency might come from different experimental duration. A previous study indicated that changes in intracellular organelles in the pineal gland could return to normal 30 days later (Logvinov et al., 2004). In our research, long-term darkness made melatonin return to its physiological level; thus, there was no difference between the two groups of rats. To further excavate the effect of melatonin, we examined levels of melatonin receptors and found decreased expression of *MTNR1A* in the darkness group of rats. These changes due to constant darkness may be in critical association with pathological alterations in PCOS development and progression.

Hyperandrogenism is a remarkable clinical feature of PCOS and could contribute to its occurrence along with insulin resistance (Condorelli et al., 2018). Accumulated studies revealed androgen excess may impair pancreatic β-cell function and enhance intra-abdominal adipocyte hypertrophy. In addition, a mouse knockout study showed that AR expression in granulosa cells is critical for normal follicular development, subsequent ovulation, and dynamic changes in ovarian steroidogenesis (Shiina et al., 2006; Astapova et al., 2019). Another notable fact is that both pre- and post-natal animal models of androgen excess have been used to study the pathophysiology of PCOS both within the ovary and with regard to overall metabolic health (Manneras et al., 2007; van Houten et al., 2012). All of aforementioned the findings implied the importance of androgen excess and its receptor in reproductive morbidity of PCOS. In our experiment, despite the appearance of hyperandrogenism, the expression of AR also significantly decreased at ZT16 in the rat ovaries of the darkness group. The expression of steroidogenesis enzyme, aromatase, became lower in the darkness group as well. Since the *Cyp17a1* level in the ovaries remained unchanged, our finding suggested that lower androgen conversion efficiency in GCs may result in PCOS-like androgen excess.

To study the role of *Mtnr1a* in hyperandrogenism of PCOS and investigate whether the pathological changes could be reversed, we applied two different rescue treatment methods following darkness intervention. One of the treatments was RMT application. As a melatonin receptor agonist, RMT primarily favors sleep initiation and resets the circadian clock to phases allowing persistent sleep. It has been used to improve primary chronic insomnia for a long time (Hardeland, 2009) and recently has been investigated in depression and anxiety treatment (Satyanarayanan et al., 2020). Recently, scientists have reported that RMT could be used to attenuate obesity and improve insulin signaling in visceral and subcutaneous white adipose tissue (Uchinaka et al., 2018). The beneficial effects of RMT shed light on the possible association between PCOS, endocrinal and metabolic disorders, and melatonin receptors. Considering that the expression of *Mtnr1a* significantly decreased in PCOS-like rats, we applied RMT lavage to rescue the influence of darkness intervention. Surprisingly, androgen excess in PCOS-like rats was remarkably relieved. Higher abundance of estradiol and improved expression of *Mtnr1a* and *Ar* in ovaries and *Cyp19a1* in GCs validated the mechanism by which decreased *Mtnr1a* contributed to PCOS occurrence in SD rats owing to continuous light deprivation. These results suggested a favorable effect of RMT in treating constant darkness-induced PCOS rats.

The second method of the rescue experiment is normal periodic circadian restoration. The alleviation of abnormal ovary morphology and hyperandrogenism demonstrated that normal circadian alignment might be another effective therapeutic method in PCOS treatment. Although circadian rhythm restoration did not influence *Mtnr1a* expression in rat ovaries, it caused higher *Cyp19a1* levels in whole ovaries. We hypothesize that light/dark rhythm restoration might contribute to androgen-excess improvement through a different way in PCOS pathologic progression rather than melatonin receptor.

Furthermore, to decipher the association between *MTNR1A* and hyperandrogenism, we made use of the human granular cell-like KGN cell line for mechanism investigation. In this part of the experiment, we found downregulated expression of *AR* and *CYP19A1* after *MTNR1A*-knockdown. A similar effect of si-*MTNR1A* was observed when the physiological concentration of testosterone was added to the culture medium. A decreased content of estradiol, an androgen conversion product, reflected a lower activity of aromatase as well. Next, we used si-*AR* to find out the function of AR in steroidogenesis. It was observed that *AR*-knockdown led to reduced aromatase expression with testosterone in the culture medium, illustrating the role of *AR* in aromatase production modulation. Finally, to figure out whether *AR* was an essential downregulation stream factor of *MTNR1A*, *MTNR1A-*knockdown along with *AR*-overexpression was conducted at the same time. Surprisingly, we found that the knockdown of *MTNR1A* did not lead to a lower aromatase protein level after *AR*-overexpression in the KGN cell line. It suggested that *AR*-overexpression could block out the effect of *MTNR1A*, and *AR* acted as an important downstream factor of *MTNR1A* in aromatase production, thus playing a vital role in the PCOS-related pathophysiological process of androgen excess.

Additionally, it is known that the melatonin receptor locates on the cell membrane, while AR functions as a nucleus factor. The signaling pathway through which *MTNR1A* regulated *AR* expression was investigated in our experiment. Lots of researchers revealed that administration of melatonin could change cAMP levels and PKA phosphorylation in cholangiocytes, human prostate epithelial cells, HEK-293 cells, and so on (Renzi et al., 2011; Tam and Shiu, 2011; Chen et al., 2014; Tao and Zhu, 2018). Also, such alterations can be blocked by the melatonin receptor antagonist, luzindole. It suggests that through binding to the receptor, melatonin plays its role *via* activation of the cAMP/PKA signaling pathway. In this experiment, we found the usage of 100 μmol/L cAMP would activate the cAMP/PKA signaling pathway and promote aromatase expression significantly. Also, cAMP-activating treatment could block the downregulation of aromatase following *MTNR1A*-knockdown. These results indicated that *MTNR1A* regulated aromatase expression through the cAMP/PKA signaling pathway.

Among the limitations of our study is the fact that we performed the experiments with the KGN cell line. Although it is considered an effective reproductive cell line alternative for human primary cells, it would be more convincing to conduct associated research *in vitro* with hGCs from female patients. The majority of our hypothesis was validated to some extent in this research although some of the findings in *in vivo* were not as same as those in *in vitro*. The research on MT1, melatonin, and downstream signal transduction pathway in PCOS needs to be further deepened, which will help to illustrate the role of circadian rhythm disorder. Additionally, despite the significant mRNA expression changes in hGCs of PCOS patients, the role of circadian rhythm misalignment in the occurrence among PCOS patients still needs to be confirmed through recruiting reproductive female volunteers into different light/dark cycles, such as female shift workers, and investigating their hormonal and reproductive changes. We assume that more epidemic information on female shift workers may be collected, and the incidence of PCOS among them may be calculated. The related molecular variations need to be confirmed in further research studies.

Together, this finding reveals that long-term circadian rhythm misalignment could result in PCOS-like hormonal and reproductive disorder, along with glucose intolerance, and MTNR1A acts as an important connecting factor between circadian rhythm dysfunction and PCOS pathophysiology. Restoring normal light/dark cycle and melatonin receptor agonist application both relieve hyperandrogenism in darkness-induced PCOS. Our study underlines that circadian rhythm misalignment potentially leads to androgen accumulation in the development of PCOS and aids to broaden the preventive strategies in clinical treatment among PCOS patients.

## Data Availability

The raw data supporting the conclusion of this article will be made available by the authors, without undue reservation.
