# Supplementary material for: Long-term environmental exposure of darkness induces hyperandrogenism in PCOS via melatonin receptor 1A and aromatase reduction
Source: Front Cell Dev Biol. 2022 Oct 24;10:954186. doi: 10.3389/fcell.2022.954186 (PMC9639332; doi:10.3389/fcell.2022.954186)
Supplement: Supplementary file 1 [file Presentation1.pdf]

Supplementary Materials

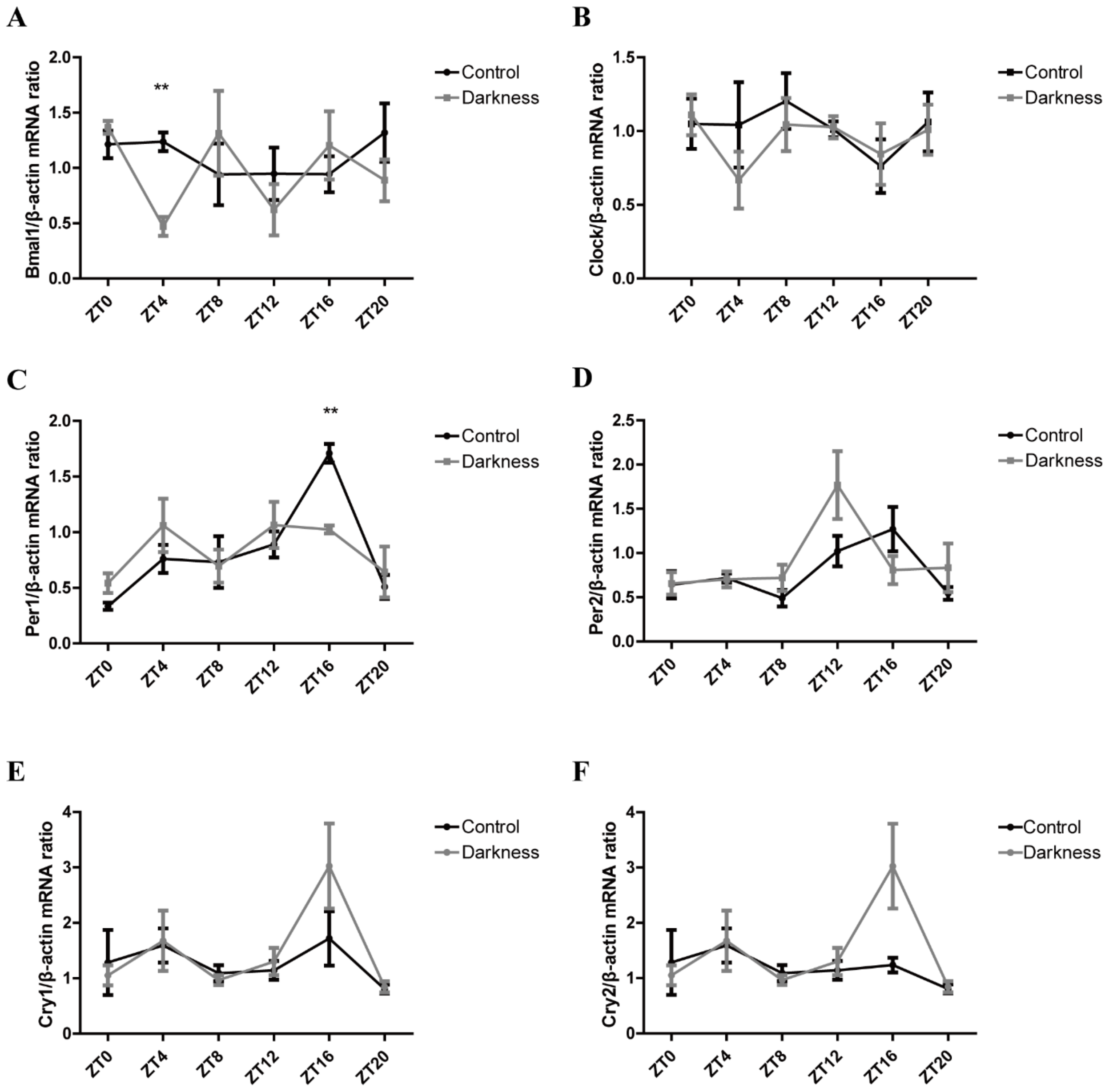

Supplementary Figure 1. Circadian gene expression pattern in rat ovaries.

(A-F) mRNA abundance of *Bmal1*, *Clock*, *Per1*, *Per2*, *Cry1*, and *Cry2* in rat ovaries, respectively. $N = 5$/group. Values are expressed as means ± SEM. Significant differences between the two groups are indicated by asterisks (*$P < 0.05$, **$P < 0.01$).

`

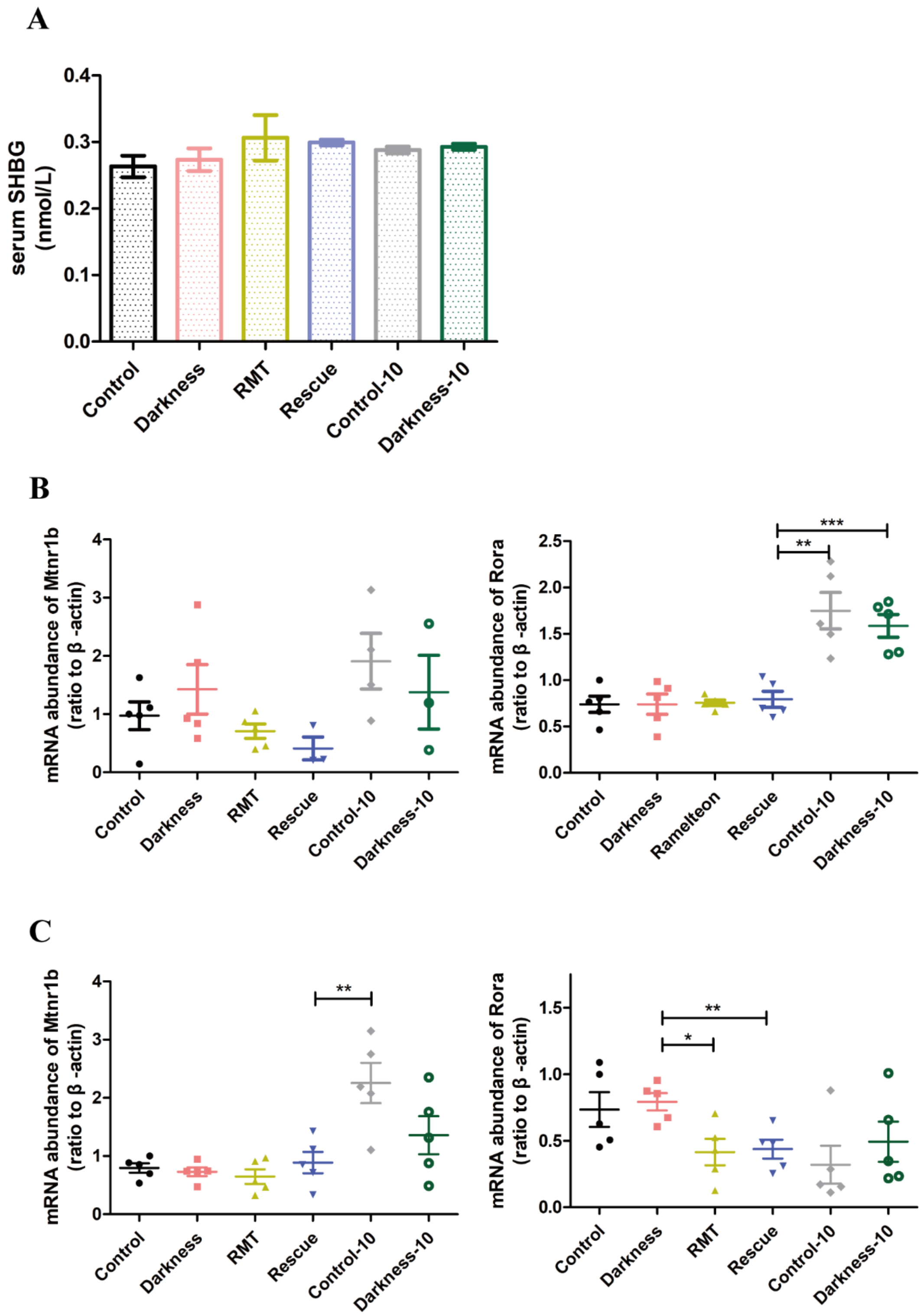

Supplementary Figure 2. SHBG and gene expression in GCs and ovary tissue of each group of rats.

(A) Serum SHBG level. **(B)** *Mtnr1b* and *Rora* mRNA abundance of GCs in each group of rats. **(C)** *Mtnr1b* and *Rora* mRNA abundance of whole ovaries in each group of rats. N = 5 or 10/group. Values are expressed as means ± SEM. Significant differences between the two groups are indicated by asterisks (*P < 0.05, **P < 0.01, ***P < 0.001).

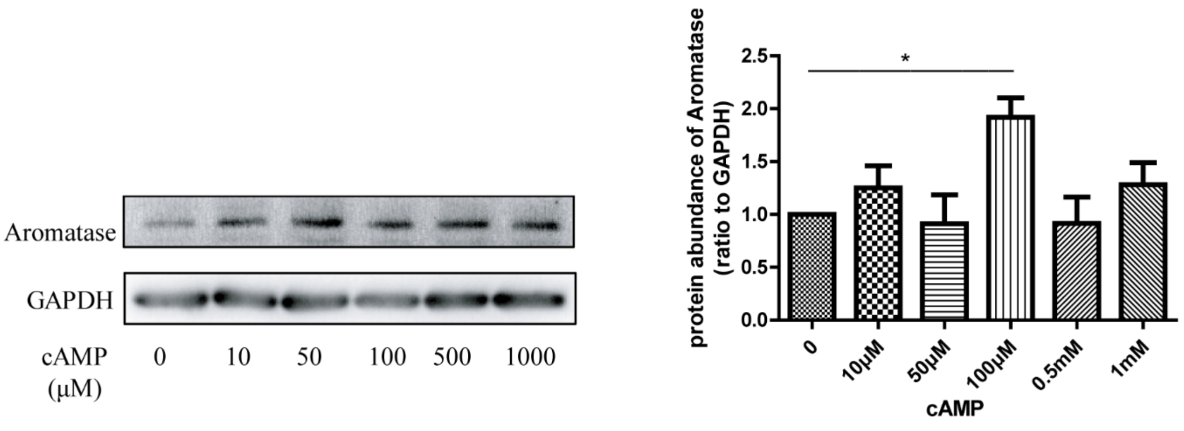

Supplementary Figure 3.

Western blot analysis (left) and quantification (right) of aromatase with 24 h-incubation of cAMP in different concentrations. Data are presented as means ± SEM. * $P < 0.05$ against control group. The result is representative of at least three independent experiments.

`

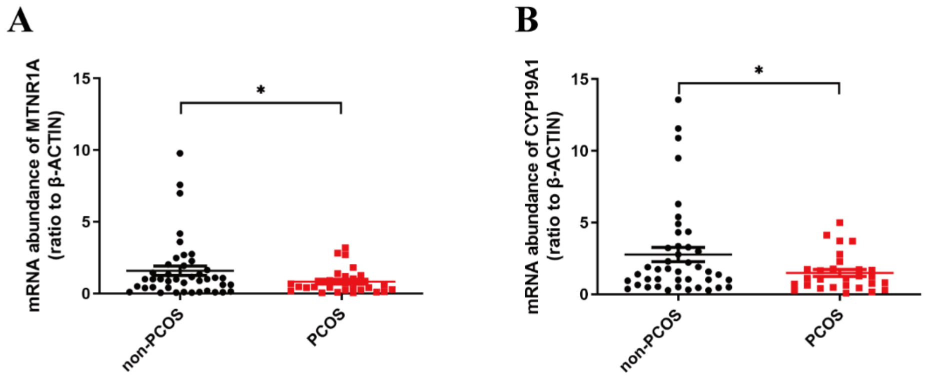

Supplementary Figure 4. Decreased mRNA levels of *MTNR1A* and *CYP19A1* in PCOS patients. Relative mRNA abundance of *MTNR1A* **(A)** and *CYP19A1* **(B)** in GCs from non-PCOS patients ($N = 42$) and PCOS patients ($N = 28$). Values are expressed as means ± SD. Significant differences between the two groups are indicated by asterisks (*$P < 0.05$).

`

Supplementary Table 1. The primer sequences.

Gene (species)	forward	reverse
Cyp17a1 (rat)	CCAGCCTGACGGACATTCTGATTC	CTGATACGCAGCACTTCTCGGATAG
Cyp19a1 (rat)	GCTACTGTCTGGGAATCGGG	GTTGCAGGCACTTCCAATCC
Ar (rat)	GACCTTATGGGGACATGCGT	TTCCCTTCCGCAGCTCTTTT
Mtnr1a (rat)	ACCGGAACTCTCCAGTACGA	GGTTTGCTGTCCGGTTTCAC
Mtnr1b (rat)	GGCCATTCTCCATGACGGTT	AGATGCACCAGTAGCGGTTG
Rora (rat)	GGGATCAAACCCGAACCCAT	GGTCTGCCACGTTATCTGCT
Bmal1 (rat)	TTTGTTTGTCGTAGGATGTGACC	CGCAGTGTCCGAGGAAGATA
Clock (rat)	CTTCCTGGTAACGCGAGAAAG	GTCGAATCTCACTAGCATCTGAC
Per1 (rat)	GATGTGGGTGTCTTCTATGGC	AGGACCTCCTCTGATTCGGC
Per2 (rat)	CAGGTTGAGGGCATTACCTCC	AGGCGTCCTTCTTACAGTGAA
Cry1 (rat)	CACTGGTTCCGAAAGGGACTC	CTGAAGCAAAAATCGCCACCT
Cry2 (rat)	CACTGGTTCCGCAAAGGACTA	CCACGGGTCGAGGATGTAGA
β-Actin (rat)	GGCCAACCGTGAAAAGATGACC	AACCCTCATAGATGGGCACAG
MTNR1A (human)	GGCCAACCGTGAAAAGATGACC	AACCCTCATAGATGGGCACAG
AR (human)	GACGACCAGATGGCTGTCATT	GGGCGAAGTAGAGCATCCT
CYP19A1 (human)	ATTGGACCCCTCATCTCCCA	TCCAGAGATCCAGACTCGCA
β-ACTIN (human)	GGGAAATCGTGCGTGACATTAAG	TGTGTTGGCGTACAGGTCTTTG

`

Supplementary Table 2. Demographic features of recruited participants.

Parameters	non-PCOS (n=42)	PCOS (n=28)
Age (years)	28.56 ± 3.08	29.19 ± 2.62
BMI (kg/m^2)	21.66 ± 2.90	23.77 ± 3.27**
Cycle length (day)	29.37 ± 1.74	50.29 ± 24.77***
Basel FSH (IU/L)	6.39 ± 1.23	5.90 ± 1.35
Basel LH (IU/L)	5.42 ± 2.17	8.66 ± 4.14***
LH/FSH ratio	0.85 ± 0.31	1.55 ± 0.82***
Basel T (nmol/L)	0.96 ± 0.37	1.36 ± 0.70*
Basel E_2 (pg/mL)	47.83 ± 27.39	53.12 ± 44.15
Basel TSH (mIU/L)	2.11 ± 1.06	1.70 ± 0.87
AMH (ng/mL)	4.17 ± 1.78	8.49 ± 4.34***
Fasting blood glucose (mmol/L)	5.11 ± 0.48	4.94 ± 0.61
Fasting insulin (μIU/mL)	7.10 ± 3.13	11.15 ± 6.65 **

All data are presented as mean ± SD. *, $P < 0.05$, **, $P < 0.01$, ***, $P < 0.001$, vs. non-PCOS group.

BMI, body mass index; FSH, follicle-stimulating hormone; LH, luteinizing hormone; E_2, estrogen; T, testosterone; AMH, anti-Müllerian hormone.

`
